# Dynamics of soil organic and inorganic carbon in the cropland of upper Yellow River Delta, China

**DOI:** 10.1038/srep36105

**Published:** 2016-10-26

**Authors:** Yang Guo, Xiujun Wang, Xianglan Li, Jiaping Wang, Minggang Xu, Dongwei Li

**Affiliations:** 1 College of Global Change and Earth System Science, Beijing Normal University, Beijing, 100875, China; 2 College of Agriculture, Shihezi University, Shihezi, 832000, China; 3 Ministry of Agriculture Key Laboratory of Crop Nutrition and Fertilization, Institute of Agricultural Resources and Regional Planning, Chinese Academy of Agricultural Sciences, Beijing, 100081, China

## Abstract

Soil inorganic carbon (SIC) and organic carbon (SOC) are important carbon reservoirs in terrestrial ecosystems. However, little attention was paid to SIC dynamics in cropland. We conducted a survey in the upper Yellow River Delta of North China Plain. We collected 155 soil samples from 31 profiles, and measured SOC, SIC and soluble Ca^2+^ and Mg^2+^ contents. Our results showed that mean SOC content decreased from 9.30 g kg^−1^ near the surface to 2.36 g kg^−1^ in 80–100 cm whereas mean SIC content increased from 10.48 to 12.72 g kg^−1^. On average, SOC and SIC stocks over 0–100 cm were 5.73 kg C m^−2^ and 16.89 kg C m^−2^, respectively. There was a significantly positive correlation (r = 0.88, P < 0.001) between SOC and SIC in the cropland. We also found that SIC had a significantly positive correlation with both soluble Ca^2+^ (r = 0.57, P < 0.01) and Mg^2+^ (r = 0.43, P < 0.05). Our study suggested that increasing SOC might lead to an increase in SIC stocks in the cropland of North China Plain. This study highlights the importance of SIC in the carbon cycle of China’s semi-arid region.

Soil carbon, including soil inorganic carbon (SIC) and organic carbon (SOC), is the largest carbon pool in the terrestrial ecosystem, thus plays an important role in the global carbon cycle and climate change^1^,^2^. The estimated global SOC storage varies within 1220–1576 Pg in the top 100 cm^3^, and the SIC storage is 700–1700 Pg^3^,^4^. There have been many studies on SOC dynamics. However, much less attention has been paid to SIC despite it is an important part for carbon sequestration and climate mitigation^5^,^6^.

Soil inorganic carbon, primarily calcium (and magnesium) carbonate, is formed mainly through the following two reactions:


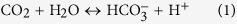



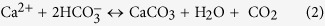


The formation of calcium carbonate is affected by soil carbon dioxide (CO_2_), pH, Ca^2+^ content and water condition. An increase in soil pH (i.e., a decrease in H^+^) would drive the reaction (1) to the right, resulting in production of HCO_3_^−^. When there is no limitation of soluble Ca^2+^/Mg^2+^, precipitation of calcium carbonate would occur in soil. On the other hand, an increase in soil CO_2_ or a decrease in soil pH would drive the reaction (2) to the left. Therefore, acidic conditions could lead to dissolution of carbonate, causing a decrease in SIC stock, whereas alkaline environment would benefit the formation of carbonate^7^. However, there are limited studies assessing how these environmental conditions regulate SIC dynamics. In particular, little information is available on the relationship of Ca^2+^/Mg^2+^ with SIC stock^8^.

Soil inorganic carbon is often found in arid, semiarid and/or semi-humid areas^5^,^9^. There were some studies of SIC dynamics in north China, which were mainly conducted in the Loess Plateau^10^,^11^, the Inner Mongolia^12^,^13^ and near the deserts of Xinjiang province^14^,^15^. Recent analyses have shown that SIC content is largely related to land use type. For example, Wang, *et al*.^15^ reported that SIC stock was much higher in cropland than in the native lands of Yanqi Basin. Some studies also showed that the SIC stock was significantly higher in cropland than in grassland, e.g., in the middle of the Hexi Corridor, Gansu, China^16^, around the Yunwu Mountain, Ningxia, China^17^, and in the Russian Chernozem^18^. These studies imply that cropland in arid and semi–arid regions may have potential for carbon sequestration as carbonate.

To date, there have been limited studies addressing the relationship between SIC and SOC, which led to inconsistent findings. Earlier analyses showed a negative relationship between SIC stock and SOC stock in northern China^19^. However, a recent study demonstrated that there was a strong positive correlation between SIC and SOC in the Yanqi Basin, northwest China^7^. Positive relationship was also reported for the soils of Canada^20^ and the USA^21^. The discrepancy may reflect the differences in climate conditions and soil properties that affect various processes in association with the transformation and accumulation of SIC and SOC^8^.

In this study, we selected a typical cropland in the upper Yellow River Delta to test the hypothesis that there is a positive relationship between SOC and SIC. The sampling area spanned both sides of the Yellow River (Fig. 1), and soils were collected from 0–20, 20–40, 40–60, 60–80 and 80–100 cm at 31 sites. The objective of this study was to investigate the implications of the Yellow River for the distributions of SOC and SIC in soil profiles and to identify the potential factors determining SIC dynamics in the typical cropland of North China Plain.

## Results

### Soil chemical properties

Soil pH in the study area ranged from 8.12 to 8.28, with high values found in the 20–60 cm layer (Table 1). Soil electric conductivity was increased from 0.24 ms cm^−1^ near the surface to 0.44 ms cm^−1^ in the 80–100 cm. Similarly, total dissolved solid also showed an increasing trend with soil depth from 576 mg kg^−1^ to 1078 mg kg^−1^. There was a small variation in the mean soluble Ca^2+^ and Mg^2+^ contents with depth, i.e., 88–93 mg kg^−1^ and 26–32 mg kg^−1^, respectively.

The spatial distributions of soluble Ca^2+^ and Mg^2+^ stocks over 0–100 cm in the sampling area were shown in Fig. 2. There were large variations in both soluble Ca^2+^ (81–191 g m^−2^) and Mg^2+^ (21–102 g m^−2^) stocks over 0–100 cm. Overall, soluble Ca^2+^ stock was apparently higher in the sites near the Yellow River than other sites far away from the Yellow River. Higher values of soluble Ca^2+^ stock were found in the southern part than in the northern part of the sampling area. Interestingly, soluble Mg^2+^ revealed a different spatial pattern, showing random high values.

### Vertical distributions of SOC and SIC

Figure 3 shows vertical distributions of SOC and SIC along each line. As expected, there was a clear decline in SOC content. Interestingly, the decline of SOC was approximately 50% from 0–20 cm to 20–40 cm, followed by a linear change with depth. On the other hand, SIC showed a slightly increasing trend over depth. There was a large similarity in terms of magnitude and vertical distribution among all lines for both SOC and SIC. However, there was a considerable variation in the surface SOC (5.86–15.16 g kg^−1^), but a large variation in SIC (7.71–21.21 g kg^–1^) of subsoil. As shown in Table 1, mean SOC content decreased from 9.3 ± 2.4 in the 0–20 cm to 4.3 ± 1.2, 3.5 ± 0.9, 2.9 ± 0.9, and 2.4 ± 0.8 g kg^–1^ in the 20–40, 40–60, 60–80 and 80–100 cm, respectively, whereas mean SIC content increased from 10.5 ± 2.6 in the 0–20 cm to ~11.7 ± 3.1 and 12.7 ± 3.8 g kg^–1^ in the 20–60 and 60–100 cm, respectively. The magnitudes and vertical distributions of SOC and SIC were similar to those in the loess profiles of northwest China^22^.

### Spatial variations of SOC and SIC stocks

Figure 4 shows the spatial distributions of SOC and SIC stocks over the upper 100 cm, which were in a range of 4.38–8.58 and 11.90–24.05 kg C m^−2^, respectively. It appeared that both SOC and SIC stocks were higher near the Yellow River than those far away from the Yellow River. Overall, SOC stock was higher in the south side of the Yellow River although there was a large variation in SOC in the southwest part of the study area. In general, SIC stock was higher in the south side of the Yellow River than in the north side, except three sites showing low value of SIC stock. Interestingly, the highest SIC values were found at the sites that showed relatively high SOC stock. On average, SIC stock (16.89 kg C m^−2^) was nearly three times of SOC stock (5.73 kg C m^−2^) over 0–100 cm in the cropland of the upper Yellow River Delta, indicating that SIC stock accounted for approximately 75% of the total carbon stock.

### The relationship between SIC and Ca^2+^ or Mg^2+^

The spatial pattern of SIC stock in this study was similar to those of soluble Ca^2+^ and Mg^2+^ stocks, especially soluble Ca^2+^ (see Figs 2 and 4). Statistical analyses indicated that SIC stock had a significantly positive relationship with soluble Mg^2+^ (r = 0.43, P < 0.05), but a stronger positive relationship with soluble Ca^2+^ (r = 0.57, P < 0.01) (Fig. 5).

## Discussion

### SOC and SIC stocks in the North China’s cropland

Our analyses showed that mean SOC stock (0–100 cm) was 5.73 kg C m^−2^ in the cropland of Zibo–Binzhou area, which was close to the values of 6.6 and 6.0 kg C m^−2^ in the cropland of Zhengzhou and Lanzhou areas, respectively (Table 2). Liu *et al*.^23^ reported slightly higher value of 7.7 kg C m^−2^ in the cropland of the Loess Plateau^23^. Our estimate was also significantly lower than the previously reported value of 8.38 kg C m^−2^ in the cropland of Hebei, the North China Plain^24^, the value of ~9.0 kg C m^−2^ in the regions of Yanqi^15^ and Urumqi^8^, Xinjing province, and that of 11.4 kg C m^−2^ in the Xilin River Basin, the Inner Mongolia^13^. The differences in SOC stock among different regions were probably due to the climate condition (such as temperature and precipitation) and land management (e.g., cropping system, fertilization and organic amendments)^22^,^25^.

The SIC stock was 16.89 kg C m^−2^ over 0–100 cm in the Zibo–Binzhou area, which was significantly higher than the reported value for the semi-arid cropland at Zhengzhou (8.14 kg C m^−2^), where soils were developed on the same parent material (i.e., the alluvial loess). The SIC stock in this study was also higher than the semi-arid cropland at Yangling (9.00 kg C m^−2^), where soil was developed on the deposited loess. A range of 12–15 kg C m^−2^ was found in the Zibo–Binzhou region for irrigated fields using the data from the Second National Soil Survey^26^, which was closed to our estimate. However, the SIC stock in our study was lower than those for the arid cropland, e.g., 20.78 kg C m^−2^ in the loess soil of Lanzhou^22^, and 51.20 kg C m^−2^ in the brown desert soil of Yanqi^15^. These large differences in SIC stock might reflect complex implications of climate conditions, soil properties and land managements for the formation of soil carbonate^12^,^13^.

### Factors influencing SIC

Carbonate (and bicarbonate) and calcium (and magnesium) are the essential elements for carbonate precipitation. Previous study has demonstrated that organic amendments in cropland of north China can significantly enhance carbonate accumulation, in particular in the subsoil^8^. Zhang, *et al*.^22^ reported that SIC stock in the loess soil of Lanzhou area was modestly higher in high fertility soil than in low fertility soil. These findings suggest that increasing soil fertility may lead to enhanced carbonate accumulation in soil profile.

To explore the impacts of soil fertility on SIC stock in the cropland of upper Yellow River Delta, we divided our data into two groups (low fertility and high fertility) using a criterion of 9.0 g kg^−1^ SOC in the 0–20 cm, which was similar to that used in the Lanzhou area by Zhang, *et al*.^22^. Our analyses showed that the mean value of SIC content ranged from 12.65 g kg^−1^ to 14.91 g kg^–1^in the high fertility soils, and from 10.07 g kg^−1^ to 12.33 g kg^−1^ in the low fertility soils (Fig. 6). The SIC stock over 0–100 cm was significantly (P < 0.05) higher in the high fertility soils (18.89 kg C m^−2^) than in the low fertility soils (16.24 kg C m^−2^).

Our analyses showed that the SIC stock was positively correlated with soluble Ca^2+^ and with soluble Mg^2+^. Given that higher soluble Ca^2+^ and Mg^2+^ were found near the Yellow River, we postulate that the sources of Ca^2+^ and/or Mg^2+^ in this area may come from the ground water and/or river water (i.e., the Yellow River). Earlier studies showed that Ca^2+^ and Mg^2+^ contents in the main channel of the lower Yellow River were 45–51 mg L^−1^ and 22–26 mg L^−1^, respectively, which were significantly higher than those (30–40 mg L^−1^ for Ca^2+^ and 6.3–14 mg L^−1^ for Mg^2+^) in the main channel of the Yangtze River^27^,^28^. The large difference in SIC stock of subsoil between Zhengzhou and Zibo–Binzhou (Table 2) despite the same soil type may be due to the accessibility of groundwater; higher groundwater table in the present study may provide more soluble Ca^2+^ and Mg^2+^ and therefore benefit the formation of SIC. In addition, there might be other sources of Ca^2+^/Mg^2+^, such as dust, fertilization, and weathering of Ca/Mg silicate minerals^5^,^8^. Further studies are needed to identify the Ca^2+^/Mg^2+^sources and to explore the mechanisms regulating SIC formation.

### The relationship between SIC and SOC

There have been inconsistent finding on the relationship between SIC and SOC. On the one hand, there was evidence that the negative relationship between SIC and SOC was found in the surface soil of North China Plain^24^ and west Loess Plateau^29^. On the other hand, a positive relationship between SIC and SOC was found in the Badan Jaran Desert, Gansu (over 0–30 cm)^16^ and in the Yanqi Basin, Xinjiang (over 0–100 cm)^8^. The negative relationship may only exist in surface layer where CO_2_ production is high due to root respiration and decomposition of SOC, which creates acidic conditions, thus leads to dissolution of soil carbonate; the positive relationship may be more common in high pH soils and without Ca/Mg limitation^7^.

Our data showed a significantly positive correlation between the SIC and SOC stocks (r = 0.88, P < 0.001) in the cropland of upper Yellow River Delta (Fig. 7). The slope for the 0–100 cm (2.87) was much greater than for the 0–30 cm (1.22). Similarly, Wang *et al*. also found that the slope reached 1.9 (over 0–100 cm) in Yanqi Basin’s cropland that had access to groundwater (containing high level of Ca^2+^/Mg^2+^)^15^. These findings imply that increasing SOC in alkaline soil can lead to an increase of SIC in subsoil if there is no limitations of calcium (and magnesium)^7^.

Table 2 illustrates that the SIC:SOC ratio was about 1.50 for 0–30 cm and 2.95 for 0–100 cm in the cropland of the upper Yellow River Delta, which was higher than those estimated in Zhengzhou and Yangling regions (0.90–0.95 over 0–30 cm and 0.96–1.27 over 0–100 cm). But our estimates for the cropland over 0–30 cm or 0–100 cm were noticeably lower than the SIC:SOC ratio in western China, i.e., 2.44–2.54 over 0–30 cm and 3.41–4.72 over 0–100 cm in Lanzhou and Yanqi. On the one hand, the inconsistency relationship between SOC and SIC may reflect the differences in the driving factors that regulate the processes associated with the transformations and accumulations of SIC in cropland. On the other hand, the robust conclusion of higher SIC in cropland’s subsoil may be related to leaching that leads to downward movement of CO_3_^2−^/HCO_3_^−^ and Ca^2+^/Mg^2+^ ions^30^.

As revealed in Fig. 8, there was no clear relationship between SOC and SIC stocks in cropland if the entire dataset was considered, in which there were various climate and soil conditions. A strong positive relationship was only seen in the Zibo–Binzhou area where soil condition, cropping system and land management were almost the same. More studies are needed to explore the relationship between SOC and SIC under various environmental conditions and different land managements, and to investigate the mechanisms regulating the accumulation of SIC in arid and semi-arid regions.

## Materials and Methods

### Characteristics of the study region

**Figure 1:**
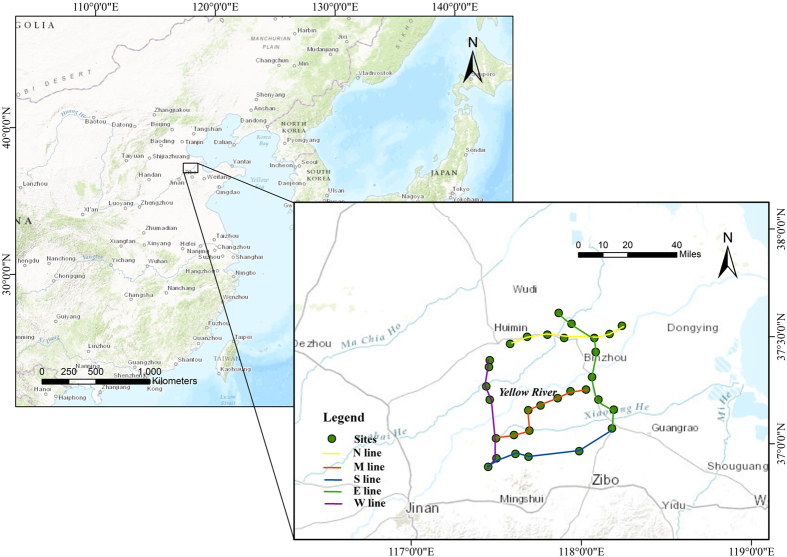
The sampling lines in the upper Yellow River Delta: north (N), middle (M), south (S), east (E) and west (W). The figure was generated by using ArcMap 10.1 (http://www.esri.com/).

**Figure 2:**
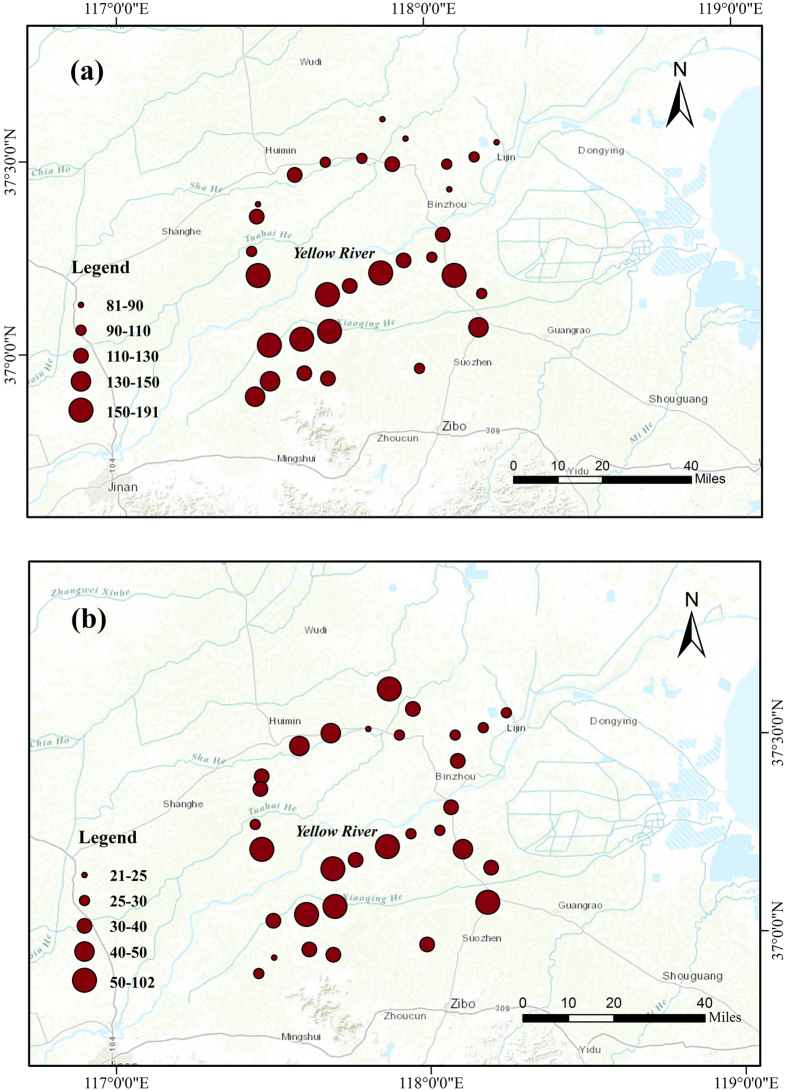
Spatial distributions of soluble Ca^2+^ (**a**) and Mg^2+^ (**b**) stocks (g m^−2^) over the 0–100 cm. The figure was generated by using ArcMap 10.1 (http://www.esri.com/).

**Figure 3:**
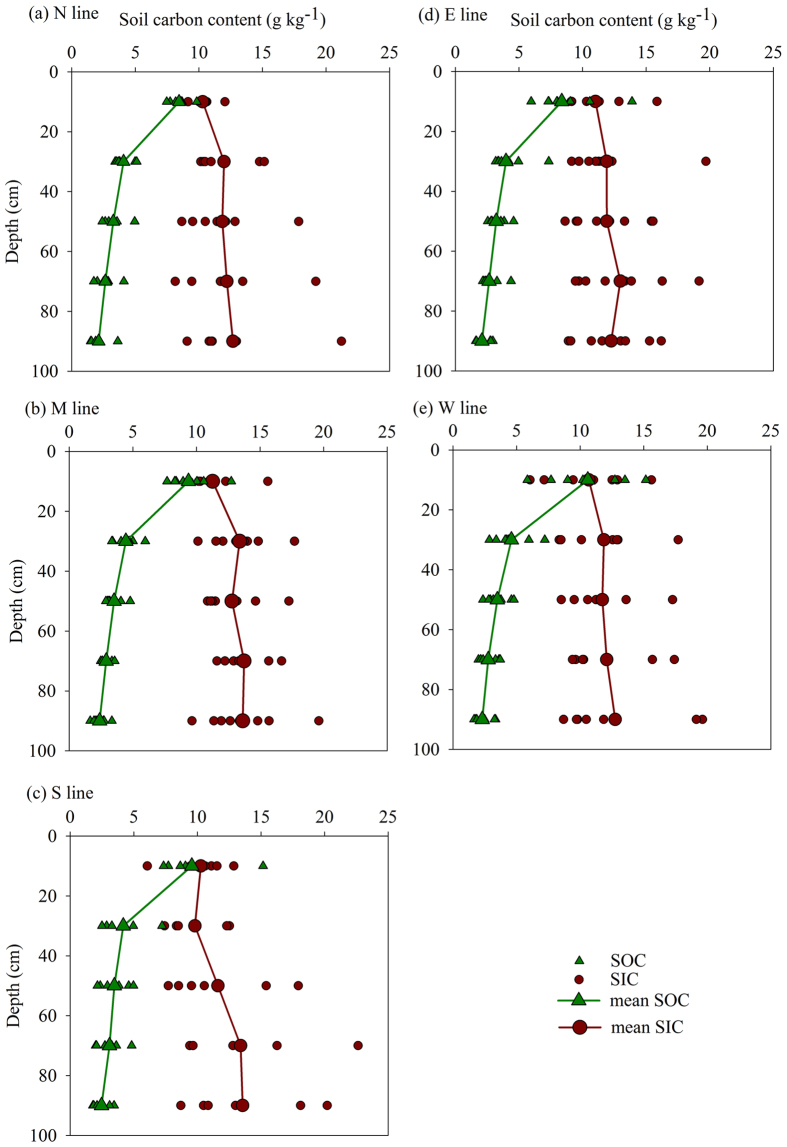
Vertical distributions of soil organic carbon content (SOC) and inorganic carbon (SIC) along the sampling lines.

**Figure 4:**
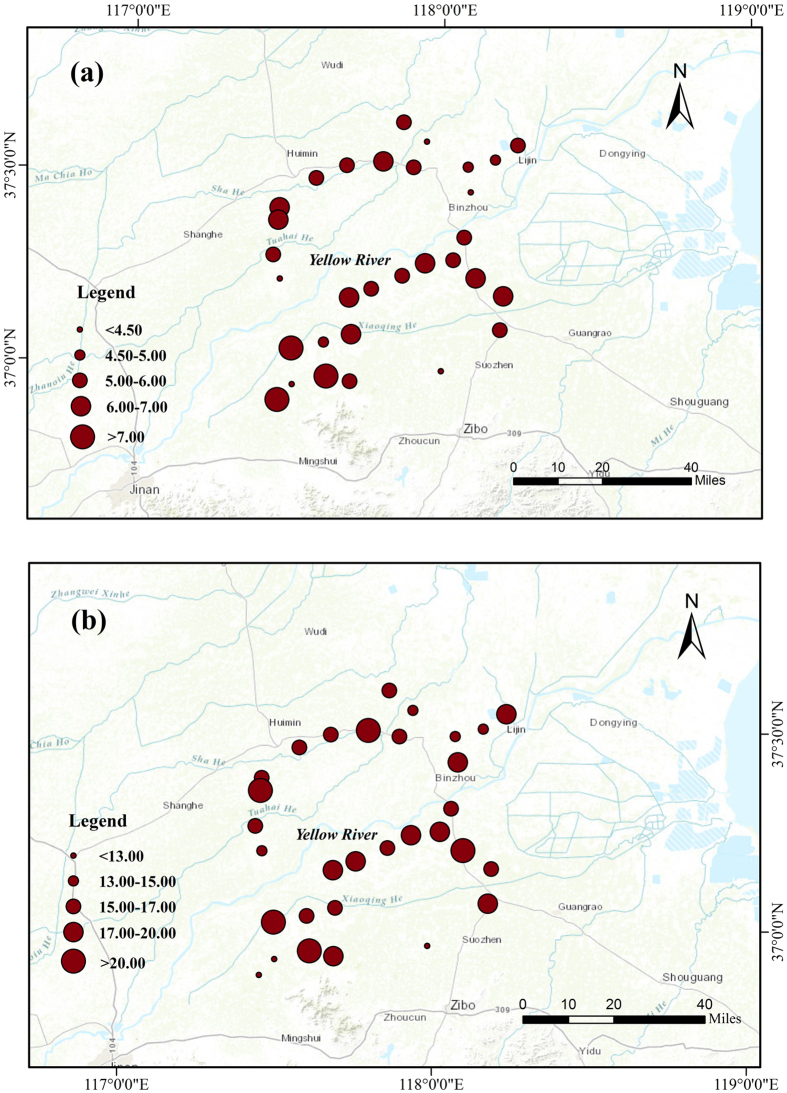
Spatial distributions of soil organic carbon (SOC) (**a**) and inorganic carbon (SIC) (**b**) stocks (kg C m^−2^) over the 0–100 cm. The figure was generated by using ArcMap 10.1 (http://www.esri.com/).

**Figure 5:**
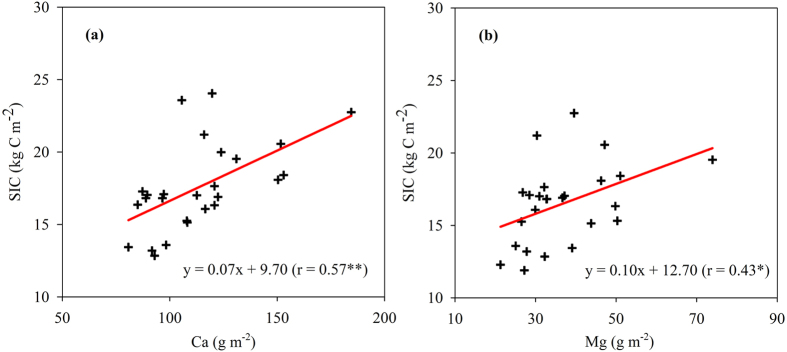
Relationship between soil inorganic carbon (SIC) and soluble ion (Ca^2+^ and Mg^2+^) stocks over the 0–100 cm. **Significant at P < 0.01; *Significant at P < 0.05.

**Figure 6:**
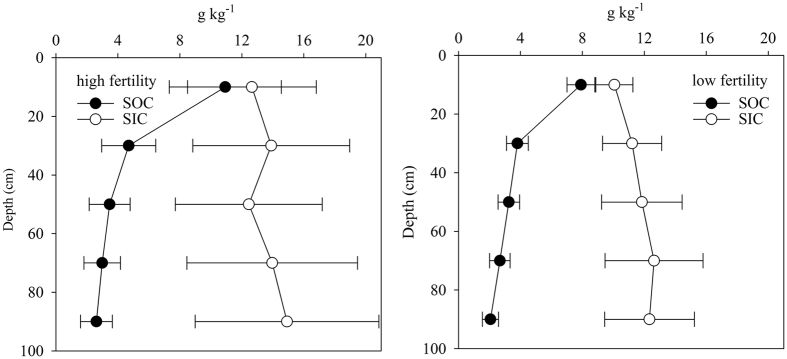
Vertical distributions of mean soil organic carbon (SOC) and inorganic carbon (SIC) for the high fertility (left) and low fertility(right) soils. The error bars indicate standard deviation. Soil profiles with SOC ≥ 9 g kg^−1^ in the upper 20 cm were regarded as high fertility, and profiles with SOC < 9 g kg^−1^ were regarded as low fertility.

**Figure 7:**
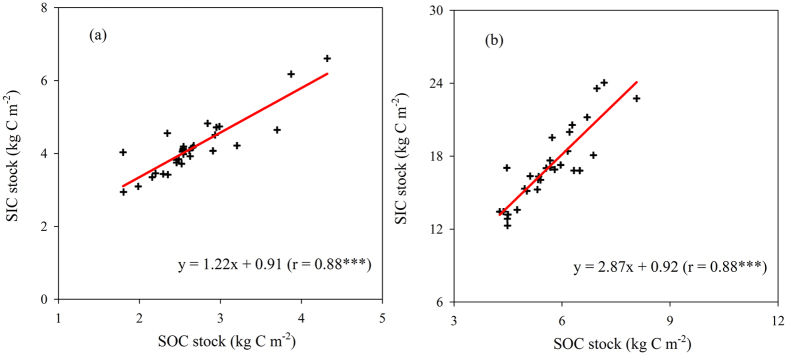
Relationship between soil inorganic carbon (SIC) and organic carbon (SOC) stocks in the 0−30 cm (**a**) and 0−100 cm soil layers (b). ***Significant at P < 0.001; **Significant at P < 0.01.

**Figure 8:**
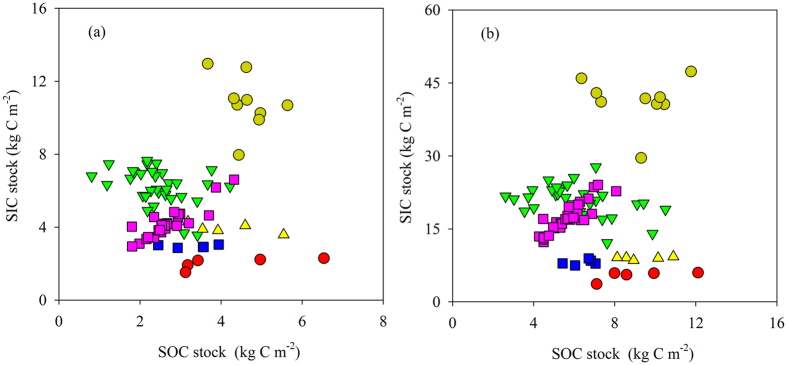
Soil inorganic carbon (SIC) verses organic carbon (SOC) stocks in the 0−30 cm (**a**) and 0−100 cm (**b**) for cropland of North China: nine profiles from Yanqi Basin (dark yellow circles), five from Urumqi (red circles), 33 from Lanzhou (green triangles), five from Yangling (yellow triangles), five from Zhengzhou (blue squares) and 31 profiles from this study (pink squares).

Our study area, in the Zibo and Binzhou City, is located in the upper Yellow River Delta (Fig. 1). The area has a typical temperate monsoon climate zone, with an annual average temperature of 13.4 °C and annual precipitation of 604 mm. Rainfall occurs mainly during the period of June–August. Thus, irrigation is often applied during other seasons, using water from the Yellow River. The soil is a loam that was developed on the Yellow River alluvial deposits, and classified as Calcaric Fluvisols^31^. Most of the land has been used for cropping for at least 50 years, mainly with a double cropping system of winter wheat (Triticum aestivum L.) and summer corn (Zea mays L.). Local farmers often apply mineral (e.g., urea and super calcium phosphate) and/or organic amendments (i.e., straw incorporation).

### Soil sampling and analyses

In order to assess the Yellow River’s influence on the distributions of SOC and SIC stocks, we sampled along five lines, in which three lines were parallel with the Yellow River and two lines across to the Yellow River. We sampled 31 soil sites in the cropland during August 2015, with sampling sites distributed as evenly as possible (about 10 km in distance between the two sites) (Fig. 1). We randomly selected four plots at each site, and collected soils at depths of 0–20, 20–40, 40–60, 60–80, and 80–100 cm, using a soil auger (5-cm diameter). Soils were air–dried, well mixed and sieved to pass a 2-mm screen for the measurements of pH, electric conductivity (EC), total dissolved solids (TDS), soluble Ca^2+^ and Mg^2+^. Soil pH and EC were measured using a soil:water (1:2.5) mixture. Soluble Ca^2+^ and Mg^2+^ were determined using a soil:water (1:5) mixture by an Atomic Absorption Spectrophotometer^4^. We used the bulk density (BD) values from the cropland in the same area, which were reported by Li, *et al*.^32^. The soil consists of 17% clay (<0.002 mm), 66% silt (0.002–0.02 mm) and 17% sand contents (0.02–2 mm). Representative sub-samples were crushed to 0.25 mm for SOC and SIC measurements. Total soil C and SOC were measured using a CNHS–O analyzer (Model EuroEA3000). For SOC measurement, 20 mg soil was pretreated with 10 drops of phosphoric acid (H_3_PO_4_) for 12 h to remove carbonate. The pretreated sample was combusted at 1020 °C with a constant helium flow carrying pure oxygen to ensure completed oxidation of organic materials. Production of CO_2_ was determined by a thermal conductivity detector. Soil inorganic carbon was calculated as the difference between total soil carbon and SOC. The analyses of total soil carbon and SOC were performed at the State Key Laboratory of Lake Science and Environment, Nanjing Institute of Geography and Limnology, Chinese Academy of Sciences.

### Calculation and statistical analyses

For each soil site, SOC and SIC stocks (kg C m^−2^) were calculated from carbon content (g C kg^−1^), BD (E_*i*_, g cm^−3^) and thickness (D_*i*_, cm):


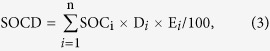



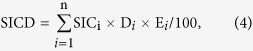


We used Fisher’s protected least significant difference (LSD) to compare SOC, SIC and soil parameters (i.e., pH, EC, and TDS) between depths. We applied Student’s *t*-test to estimate significance for the difference in SIC stock between high fertility and low fertility. Linear regression analyses were carried out to evaluate the relationship of SIC with SOC or soluble Ca^2+^/Mg^2+^. These analyses were performed using Sigmaplot (version 12.5) and Arcgis (version 10.1).

## Additional Information

**How to cite this article**: Guo, Y. *et al*. Dynamics of soil organic and inorganic carbon in the cropland of upper Yellow River Delta, China. *Sci. Rep.* **6**, 36105; doi: 10.1038/srep36105 (2016).

**Publisher’s note:** Springer Nature remains neutral with regard to jurisdictional claims in published maps and institutional affiliations.

## Figures and Tables

**Table 1:** Mean values (standard deviations) of soil pH, electric conductivity (EC), total dissolved solids (TDS), soluble Ca^2+^ and Mg^2+^, soil organic carbon (SOC) and inorganic carbon (SIC) in different soil layer, averaged over 31 sampling sites.

Soil layer depths (cm)	pH	EC (mS cm^−1^)	TDS (mg kg^−1^)	Ca^2+^ content (mg kg^−1^)	Mg^2+^ content (mg kg^−1^)	SOC content (g kg^−1^)	SIC content (g kg^−1^)
0–20	8.12 (0.22) b	0.24 (0.11) d	576 (267) d	90 (35) a	26 (10) a	9.30 (2.37) a	10.48 (2.58) b
20–40	8.28 (0.22) a	0.27 (0.15) cd	647 (330) cd	85 (36) a	27 (17) a	4.30 (1.16) b	11.72 (3.01) ab
40–60	8.24 (0.22) ab	0.33 (0.17) bc	813 (429) bc	85 (29) a	28 (14) a	3.45 (0.90) b	11.64 (3.20) ab
60–80	8.17 (0.16) b	0.41 (0.19) ab	992 (498) ab	89 (35) a	29 (16) a	2.91 (0.91) bc	12.60 (3.83) a
80–100	8.15 (0.17) b	0.44 (0.20) a	1078 (508) a	91 (40) a	31 (18) a	2.36 (0.83) c	12.72 (3.88) a

Mean values followed by the same letter are not significantly different at P < 0.05 (LSD test).

**Table 2:** Soil organic carbon (SOC), inorganic carbon (SIC) stocks and the SIC:SOC ratio in typical croplands of North China.

Region	The annual precipitation (mm)	Soil types	pH	SOC stock (kg C m^−2^)		SIC stock (kg C m^−2^)		The SIC/SOC ratio		Reference
				0–30 cm	30–100 cm	0–30 cm	30–100 cm	0–30 cm	0–100 cm	
Yanqi (n = 9)	80	Brown Desert Soil	8.2	4.53 ± 0.52	4.38 ± 1.69	11.07 ± 1.45	31.3 ± 7.42	2.44	4.72	Wang *et al*.^15^
Urumqi (n = 5)*	299	Gray Desert Soil	8.1	4.25 ± 1.49	4.90 ± 0.65	2.03 ± 0.31	3.38 ± 0.77	0.48	0.59	Wang *et al*.^8^
Lanzhou (n = 33)	300	Loess Soil	8.9	2.46 ± 0.74	3.64 ± 1.44	6.24 ± 0.99	14.54 ± 2.39	2.54	3.41	Zhang *et al*.^22^
Yangling (n = 5)*	585	Manural Loess Soil	8.6	4.16 ± 0.93	5.18 ± 0.26	3.94 ± 0.27	5.06 ± 0.19	0.95	0.96	Wang *et al*.^8^
Zhengzhou (n = 5)*	641	Fluvo-aquic Soil	8.3	3.29 ± 0.59	3.31 ± 0.28	2.95 ± 0.08	5.19 ± 0.39	0.9	1.27	Wang *et al*.^8^
Zibo-Binzhou (n = 31)	600	Fluvo-aquic Soil	8.2	2.70 ± 0.64	3.03 ± 0.64	4.06 ± 0.81	12.83 ± 2.80	1.50	2.95	This study

^*^Each site collected in 2002 and 2009 with three replicates.

## References

[b1] Eswaran H., Vandenberg E. & Reich P. Organic-Carbon in Soils of the World. Soil Sci Soc Am J 57, 192–194 (1993).

[b2] Lal R. Soil carbon sequestration impacts on global climate change and food security. Science 304, 1623–1627, 10.1126/science.1097396 (2004). 15192216

[b3] Eswaran H. . In Global Climate Change And Pedogenic Carbonates (eds Lal R., Kimble J. M., Eswaran H. & Stewart B. A.). 15–27 (CRC press, 2000).

[b4] Batjes N. H. Total carbon and nitrogen in the soils of the world. Eur J Soil Sci 47, 151–163 (1996).

[b5] Lal R. & Kimble J. M. InGlobal Climate Change And Pedogenic Carbonates (eds Lal R., Kimble J. M., Eswaran H. & Stewart B. A.). 1–14 (CRC Press, 2000).

[b6] Zheng J. . Perspectives on studies on soil carbon stocks and the carbon sequestration potential of China. Chinese Science Bulletin 56, 3748–3758 (2011).

[b7] Wang X. J. . Carbon accumulation in arid croplands of northwest China: pedogenic carbonate exceeding organic carbon. Sci Rep-Uk 5 (2015). 10.1038/srep11439 PMC4473677 26091554

[b8] Wang X. J. . Fertilization enhancing carbon sequestration as carbonate in arid cropland: assessments of long-term experiments in northern China. Plant Soil 380, 89–100 (2014).

[b9] Yang L. F. & Li G. T. Advances in research of soil inorganic carbon. Chinese Journal of Soil Science 42, 986–990 (2011).

[b10] Tan W. F. . Soil inorganic carbon stock under different soil types and land uses on the Loess Plateau region of China. Catena 121, 22–30, 10.1016/j.catena.2014.04.014 (2014).

[b11] Chang R. Y., Fu B. J., Liu G. H., Wang S. & Yao X. L. The effects of afforestation on soil organic and inorganic carbon: A case study of the Loess Plateau of China. Catena 95, 145–152 (2012).

[b12] Wang Y. G., Li Y., Ye X. H., Chu Y. & Wang X. P. Profile storage of organic/inorganic carbon in soil: From forest to desert. Sci Total Environ 408, 1925–1931 (2010). 20129647 10.1016/j.scitotenv.2010.01.015

[b13] Wang Z. P. . Soil organic and inorganic carbon contents under various land uses across a transect of continental steppes in Inner Mongolia. Catena 109, 110–117, 10.1016/j.catena.2013.04.008 (2013).

[b14] Feng Q., Endo K. N. & Guodong C. Soil carbon in desertified land in relation to site characteristics. Geoderma 106, 21–43 (2002).

[b15] Wang J. P., Wang X. J., Zhang J. & Zhao C. Y. Soil organic and inorganic carbon and stable carbon isotopes in the Yanqi Basin of northwestern China. Eur J Soil Sci 66, 95–103 (2015).

[b16] Su Y. Z., Wang X. F., Yang R. & Lee J. Effects of sandy desertified land rehabilitation on soil carbon sequestration and aggregation in an arid region in China. J Environ Manage 91, 2109–2116 (2010). 20630649 10.1016/j.jenvman.2009.12.014

[b17] Liu W., Wei J., Cheng J. & Li W. Profile distribution of soil inorganic carbon along a chronosequence of grassland restoration on a 22-year scale in the Chinese Loess Plateau. Catena 121, 321–329, 10.1016/j.catena.2014.05.019 (2014).

[b18] Mikhailova E. A. & Post C. J. Effects of land use on soil inorganic carbon stocks in the Russian Chernozem. J Environ Qual 35, 1384–1388 (2006). 16825458 10.2134/jeq2005.0151

[b19] Pan G. X. & Guo T. In Global Climate Change And Pedogenic Carbonates (eds Lal R., Kimble J. M., Eswaran H. & Stewart B. A.). 135–148 (CRC Press, 2000).

[b20] Landi A., Mermut A. R. & Anderson D. W. Origin and rate of pedogenic carbonate accumulation in Saskatchewan soils, Canada. Geoderma 117, 143–156 (2003).

[b21] Stevenson B. A., Kelly E. F., McDonald E. V. & Busacca A. J. The stable carbon isotope composition of soil organic carbon and pedogenic carbonates along a bioclimatic gradient in the Palouse region, Washington State, USA. Geoderma 124, 37–47 (2005).

[b22] Zhang F., Wang X. J., Guo T. W., Zhang P. L. & Wang J. P. Soil organic and inorganic carbon in the loess profiles of Lanzhou area: implications of deep soils. Catena 126, 68–74, 10.1016/j.catena.2014.10.031 (2015).

[b23] Liu Z. P., Shao M. A. & Wang Y. Q. Effect of environmental factors on regional soil organic carbon stocks across the Loess Plateau region, China. Agriculture, Ecosystems & Environment 142, 184–194 (2011).

[b24] Huang B., Wang J. G., Jing H. Y. & Xu S. W. Effects of Long- term Application Fer tilizer on Carbon Stor age in Calcar eous Meadow Soil. Journal of Agro-Environment Science 25, 161–164 (in Chinese) (2006).

[b25] Wu H., Guo Z. & Peng C. Land use induced changes of organic carbon storage in soils of China. Global Change Biol 9, 305–315 (2003).

[b26] Wu H., Guo Z., Gao Q. & Peng C. Distribution of soil inorganic carbon storage and its changes due to agricultural land use activity in China. Agriculture, Ecosystems & Environment 129, 413–421, 10.1016/j.agee.2008.10.020 (2009).

[b27] Cheng J. S., Wang F. Y., Meybeck M., He D. W., Xia X. H. & Zhang L. T. Spatial and temporal analysis of water chemistry records (1958–2000) in the Huanghe (Yellow River) basin. Global biogeochemical cycles 19, 2299–2310 (2005).

[b28] Chen J. S., Wang F. Y., Xia X. H. & Zhang L. T. Major element chemistry of the Changjiang (Yangtze River). Chemical Geology 187, 231–255 (2002).

[b29] Zeng J., Guo T. W., Bao G. X., Wang Z. & Sun J. H. Effections of soil organic carbon and soil inorganic carbon under long-term fertilization. Soil and Fertilizer Sciences in China 2, 11–14 (in Chinese) (2008).

[b30] Li G. T., Zhang C. L. & Zhang H. J. Soil inorganic carbon pool changed in long-term fertilization experiments in north China plain. World Congress of Soil Science: Soil Solutions for A Changing World, 220–223 (2010).

[b31] Shi Y. . Integrated management practices significantly affect N2O emissions and wheat–maize production at field scale in the North China Plain. Nutr Cycl Agroecosys 95, 203–218 (2013).

[b32] Li Y., Zhang H. B., Chen X. B., Tu C. & Luo Y. M. Gradient distributions of nitrogen and organic carbon in the soils from inland to tidal flat in the Yellow River Delta. Geochimica 43, 338–345 (2014).

